# 2021 Veterinary Medicine and Science Reviewer List

**DOI:** 10.1002/vms3.743

**Published:** 2022-01-25

Abdalla, Mohamed Abdelsalam

Abdel‐Daim, Mohamed

Abdollahifar, Mohammad‐Amin

Abdoshah, Mohammad

Abdullah, S

Abdullah, S.

Abebe, A

Addo, Seth

Afshan, Kiran

Agba, Kondi

Agga, Getahun

Agnew, Dalen

Agut, Amalia

Ahaduzzaman, Md

Ahlawat, Sonika

Ahmad, Ali Anwar

Ahmad, Iqbal

Ahmadi, Elham

Ahmadipour, Behnam

Ahmed, Dildar

Ahmed, Heba A

Ahmed, Hiwa

Ahmed, Shahana

Aivelo, Tuomas

Akbar, Ali

Akbar, Haji

Aktas, Munir

Al Aiyan, Ahmad

Al‐Iraqi, Osama

Al‐Mahmood, Omar

Alam, MZ

Albuquerque, ACA

Alexander, B

Algammal, Abdelazeem

Alhussien, MN

Ali, Abdelwahid

Ali, Destaw

Ali, Medhat

Ali, Saddam

Ali, Shahzad

Alic, Amer

Allam, Ahmad

Alsafy, Mohamed

Altuğ, Nuri

Amaral, Rodrigo

Ambrósio, Carlos Eduardo

Ameni, Gobena

Amer, M. M.

Amer, Shimaa A.

Amiri Ghanatsaman, Zeinab

Ammar, S

An, Dong‐Jun

Anandan, S

Andersson, Leif

Andres, Damian de

Anis, Anis

Anselmi, Carlo

Anson, A.

Anwar, Firoz

Appolinario, Camila

Arabestani, Mohammad Reza

Arai, K.

Arcelli, R.

Arlt, Sebastian

Armengou, Lara

Armstrong, Rob

Arrebola Molina, Francisco

Arslan, Cavit

Arslan, Handan Hilal

Aslani, Mohammad Reza

Ateş, Can

Attia, Youssef A.

Avicor, S. W.

Awan, Gulam Yaseen

Azmi, Kifaya

Babaei, M

Babiuk, Shawn

Bacek, Lenore

Badiei, Khalil

Badirzadeh, Alireza

Bae, Seulgi

Bahloul, chokri

Bailey, James

Bajcsy, Arpad

Bakhshi, Hasan

Bakker, Douwe

Bakos, Zoltan

Baldrighi, Julia

Balsa, Ingrid

Bamouh, Z

Banovic, Frane

Barhoumi, Kamel

Barlow, John W.

Barrot, A.

Bartlow, A

Barua, Himel

Basiratpour, Asal

Basler, C.

Bastos, Armanda

Baymakova, Magdalena

Bayssa, M

Beck, Cécile

Becker, Daniel J.

Becker, Jens

Beja‐Pereira, Albano

Benato, Livia

Benson, C.

Berghaus, Londa J.

Berman, J.

Bermejo‐Alvarez, Pablo

Bernabucci, Umberto

Bernard, John K.

Bernigau, Dora

Bertelloni, Fabrizio

Bertin, Francois‐Rene

Beths, Thierry

Beyer, Wolfgang

Beyi, Ashenafi F.

Bezdíček, Jiří

Bhandari, Shivish

Bharti, Vijay

Biswas, R

Biswas, Suman

Blackall, Patrick J.

Blanda, Valeria

Blasdell, Kim

Boag, A.

Boateng, Joshua

Boeri, EJ

Boqvist, Sofia

Boroffka, Susanne

Boscan, Pedro

Boudreau, E.

Boudreaux, Bonnie

Boutet, Bruno

Bouzari, Majid

Boveland, Shannon

Brady, Rachel

Brenig, Bertram

Bron, Bieneke

Bronzo, Bronzo VB

Brooke, Greg

Brooks, Kelly

Brunetto, Márcio

Buabeid, Manal

Bueno, Irene

Buhr, Tony L.

Burt, Felicity

Burton, Jenna

Busse, Claudia

Byaruhanga, C

Byers, Kaylee

Cadet, Jean

Calero‐Bernal, Rafael

Callealta, Isabel

Calvo, Jorge

Camero, Michele

Campion, D.

Canadas, Ana

Cao, Xingyuan

Capitan, R.

Cardinali, Martina

Cardoso, M

Carmena, David

Carpenter, Ann

Carr, John

Carretón, Elena

Carvalho da Costa, Marliete

Carvalho, P.

Carvalho, S.

Caspe, Gaston

Castillo, V.

Catchpole, Brian

Ceballos‐Marquez, Alejandro

Celeghini, Eneiva Carla

Celik, Ozgur

Cenariu, Mihai

Cerbu, Constantin Gheorghe

Cesari, A

Chaintoutis, Serafeim

Chako, Clemence

Chakravarti, Soumendu

Chalmeh, Aliasghar

Chan, C.

Chanjula, Pin

Chaves, Alexandre Vieira

Chavoshi, Hosein

Chay‐Canul, A

Cheema, R. S.

Chelgerdi, Mohammad

Chen, Dongmei

Chidumayo, Nozyechi

Chies, Jose Artur Bogo

Chinikar, Sadegh

Chiti, L.

Chitty, John

Cho, Jongki

Choe, Seongjun

Choi, K‐S

Chung, Jin‐Young

Ciğerci, Ibrahim Hakkı

Clauss, Marcus

Clementino, Inacio Jose

Clercx, C.

Çolakoğlu, Esra

Collins, S.

Colombo, Martina

Colwell, DD

Corgozinho, Katia Barao

Coronado, Liami

Cossaboom, C.

Costa‐Bortoliero, C

Costa, Giovanna

Cota, J

Covre, N

Coyne, M.

Credille, Brent

Cridge, H.

Crispo, Manuela

Crole, Martina Rachel

Croome, Kristopher P.

Cueto, MI

Cuevas, Élfego

Cui, Qingxin

Cui, Yongyong

Cummings, Kevin

Cutrignelli, Monica Isabella

Czopowicz, Michał

Dabrowski, Roman

Dalfardi, Behnam

Dalirijoupari, Morteza

Dall Agnol, Alais

Dang, De Xin

Dang, Ruihua

Daniels, Simon

Dantas, Antonio F. M.

Darbro, Jonathan

Darroudi, Majid

Darwish, W

Das, Meena

Dasgupta, Swagata

Dashti, Alejandro

Daud, A

Daugschies, Arwid

Davies, Peers

Davoodi, Farshid

Davudypoor, Somaye

Dawood, Mahmoud

Dayan, André

De las Heras, M

De Souza, Diogo Benchimol

Debelo, Motuma

Decloedt, Annelies

DeFrancesco, T. C.

Dermauw, Veronique

Dersch, Rachel

Dewhurst, Richard

Di Giuseppe, Marco

Di, Ran

Dias, Joana N R

Dias, Julia

Dicker, S.

Dierenfeld, Ellen S.

Dietze, Klaas

Dimitrov, Kiril M.

Dione, Michel

Dixit, AK

Dobos, Attila

Dobromylskyj, Melanie J.

Dos Santos, L.

Douadi, Benacer

Dow, Steven

Drake, Elizabeth R.

Drillich, Marc

Duha S. Ahmed

Dundon, William G

Dunham‐Cheatham, Sarrah M

Dunowska, M.

Duran‐Struuck, Raimon

Durrani, Aneela Zameer

Dye, Charlotte

Dyson, S.

Eghbalsaied, S

Egner, B.

Tadesse Eguale

Eisenschenk, Melissa

El Basuini, Mohammed F.

El Goulli, Amel

El‐Din, Eman Ahmed Alaa

El‐Hage, Charles

El‐Sabrout, Karim

Elbestawy, A. R.

Elliott, James

Elmas, Muammer

Elmetwally, Mohammed A.

Elnasharty, M

Elsenousi, Abdussalam

Elzo, Mauricio A

Emiliano, Lasagna

Engels, Geraldine

Ergunay, Koray

Eriksen, T.

Eschbaumer, Michael

Eser, Ferda

Esmailizadeh, Ali

Essa, Eman

Estrada‐Reyes, Zaira M.

Fabre, S

Fajt, Virginia

Falgenhauer, Linda

Fan, Zhiyong

Fang, Xing‐Tang

Farahpour, Mohammad Reza

Farrell, Kate S.

Farrelly, John

Farzad‐Mohajeri, Saeed

Fashae, Kayode

Fast, Christine

Feodorova, Valentina

Ferguson, Sylvia

Fernandes, Adeline

Fernandez‐Ruiz, Javier

Ferré‐Dolcet, Luis

Fietz, Daniela

Fietz, Simone

Finding, Elizabeth

Fischer‐Tenhagen, Carola

Fischer, Kerstin

Fischman, Maria

Fish, Eric

Flowers, W. L.

Forneris, Andrea

Forth, Jan Hendrik

Forwood, Daniel

Fouad, A. M.

Fox, Edward M.

Franz‐Odendaal, Tamara A.

Freitas‐de‐Melo, Aline

Freuling, Conrad

Froes, Tilde Rodrigues

Frouhar, Ali

Fthenakis, G

Fuentes, M

Furbeck, Dr. Rebecca

Furlan, João Pedro Rueda

Gäbel, Gotthold

Gabler, Christoph

Gabner, Simone

Gaillard, Charlotte

Galal Mansour, Shimaa

Galay, Remil

Gallardo, Carmina

Ganjei, Justin

Gariglio Xavier, Rafael

Gashaw, Tadele

Gelalch, Benti

Genç, Serdar

Gentry, Christina

Georgiev, Plamen

Geschwind, Jean‐Francois H.

Gethmann, Jörn

Ghaneialvar, Hori

Ghanem, Nasser

Gharbi, Mohamed

Ghimire, Tirth Raj

Ghosh, Abhrajyoti

Ghosh, Sharmila

Giaretta, Paula

Gibbons, James

Gimmel, Angela

Giorgi, Mario

Gitao, George

Gnat, Sebastian

Gokbulut, Cengiz

Gomez, M.

González, José A.

Gordon, Ira

Gordon, Stuart J. G.

Gorlov, Ivan Fiodorovich

Gou, Xiao

Gough, Sarah

Graham, John

Grahn, Bruce

Grant, Caitlin

Grant, Irene R

Grazul‐Bilska, Anna

Griffiths, Kate

Gross, Josef J.

Gruber, Erika

Grünberg, Walter

Grützner, Niels

Guerra Segundo, Daniel

Guerra, Maria Madalena Pessoa

Gugjoo, Mudasir Bashir

Guirola, Jose A.

Güler, Ali

Gumi, Balako

Gunasekaran, T.

Guo, Shi‐Ning

Gutierrez‐Quintana, Rodrigo

Gwida, Mayada

Hadziomerovic, N

Haile, Tamirat

Haji‐Hajikolaei, Mohammad‐Rahim

Hajipour, Nasser

Hallowell, Gayle

Ham, Kat

Hamdi, J

Hamor, R.

Hamouda, Awatef

Han, Jianlin

Hananeh, Wael

Handl, Stefanie

Hanninen, Marja‐Liisa

Harder, Timm

Hardjo, Sureiyan

Hare, JM

Harmon, David

Harris, Adam

Harvey, J.

Hassan, Mohammad Mahmudul

Hassanin, Ola

Hassim, Ayesha

Hazman, Omer

Hedayati, Mahdi

heidari, zahra

Heikkila, A.M.

Heilmann, Romy

Helmy, Y.

Henrique, F

Henson, Fran

Hergert, Melinda

Hernandez, J.

Hessheimer, Amelia J.

Hirsbrunner, Gaby

Hisasue, Masaharu

Hoddinott, Katie

Holst, Bodil Strom

Hopster‐Iversen, Charlotte

Hoque, Mehboob

Hosie, Margaret

Hossain, Mohammed Kawser

Ghavi Hossein‐Zadeh, Navid

Hosseinnezhad, Hedayat

Hosseinzadeh, Saeid

Hristov, A. N.

Hryciw, Deanne H.

Hsieh, Jen‐Fang

Hu, Defu

Huang, Kang

Huang, Shianglin

Hunt, J.

Hyatt, Doreene

Iamsaard, Sitthichai

Ibrahim, Ahmed

Ichinohe, Tom

Imani Rastabi, Hadi

Innerå, Marie

Interlandi, C.

Isgren, C. M.

Ishihara, Kanako

Islam, Ausraful

Islam, Md.

Islam, Mohammad Rafiqul

Ismaeel, Salam

Ivanković, Ante

Jafari, Ali

Jaffey, J. A.

Jain, A

Jalali, Seyedeh Missagh

Janssens, Geert

Jarikre, TA

Jayalakshmi, K

Jeffery, Nick D.

Ji, Rodany

Jiang, Shijin

Jianlin, Han

Jimenez, Isabel

Jodelko, Agnieszka

Jogi, J

Johannisson, Anders

Johnson, Wayne

Jonker, Annelize

Jordan, T.

Joy, Shiny

Jung, Dong In

Kabir, S. M. Lutful

Kacar, S.

Kadegowda, Anil

Kamel, Mohamed

Kanchi, Suvardhan

Kanengoni, Arnold T.

Kang, Byeon‐Teck

Kang, Li

Kar, Sirri

Kaszak, Ilona

Keller, Markus

Kennedy, Katy

Kent, Michael

Kerdsin, Anusak

Keskin, Dilek

Ketzis, Jennifer

khajali, fariborz

Khalil, Wael

Khamesipour, Faham

khan, Javed Masood

Khan, Muhammad

Khan, Wali

Khanam, Surrya

Kharrati‐Koopaee, Hamed

Khatoon, Rukhsana

Khol, Johannes

Khosravi, Mohammad

Kifle, Temesgen

Kim, Geon A.

Kim, Hye Kwon

Kim, In Ho

Kirkland, Peter

Kiupel, Matti

Kiyimba, Frank

Klein, Terry A.

Klobusicky, Pavol

Knell, Sebastian C.

Köhler, Heike

Koziol, J

Krajewska‐Wędzina, Monika

Krutova, Marcela

Kul, Ertuğrul

Kumar, Amit

Kumar, Ashok

Kumar, Dr. Pankaj

Kumsa, Bersissa

Kutzler, Michelle

Kvidea, Sara

Kwenti, Tebit

Kwiecien, Eduardo

Laameche Foudil

Ladlow, Jane

Lahiani, J.

Jeff Lakritz

Lamagna, Barbara

Lancioni, Hóvirág

Landgraff Chrystal

Larson, Laurie

Lattimer, Jimmy

Lauzi, S.

Laven, Richard

Lavergne, Sidonie

Lawson, C.

Lazarus, D.D.

Lederman, Zohar

Lee, AM

Lee, C.

Lee, Diane

Lee, Keun Hwa

Lee, Kuen

Lejeune, M.

Lekcharoensuk, Porntippa

lesmana, ronny

Lewis, Daniel D.

Li, Diyan

Li, Hegang

Li, J. L.

Li, Jing Lei

Li, Meng

Li, Yong

Liao, Jingqiu

Liaqat, Iram

Lichoti, Jacqueline

Lilliehook, Inger

Lin, Chao‐Nan

Lin, Chen‐Si

Lin, Chung‐Tien

Lin, Mao

Lindemann, M. D.

Liu, Jun

Liu, Lei

Liu, Maojun

Liu, Penggang

Liu, Qinfang

Liu, Qingyou

Liu, T

Liu, Xiao

Liuti, Tiziana

Lockwood, Randall

Lodde, Valentina

Long, Chunlin

Loria, Guido Ruggero

Lotfi, A.

Lotfollahzadeh, Samad

Loureiro, Ana Paula

Lu, Chi‐Cheng

Luca, Marco Di

Luini, Mario Vittorio

Lukanov, Hristo

Luna‐Palomera, C.

Luo, Xugang

Lüttgenau, Johannes

Ma, Zhijie

Mabelebele, M.

Machtinger, Erika

Mack, Sarah

Macri, Francesco

Madeira de Carvalho, Luís

Magnusson, Ulf

Magyar, Tibor

Mahdavi, Amir

Mahmoud, Karima

Malik, Richard

Mamlouk, A

Manger, Paul R.

Marcondes, Marcos I.

Martin, Adam

Martins, W

Mathewos, Mesfin

Mavrommatis, A

May, Anna

Mazrouei Sebdani, Mohammad

McOrist, Steven

Meichner, Kristina

Melaku, Achenef

Menzies‐Gow, Nicola

Mertens‐Scholz, K

Mesic, Zeljka

Metselaar, Matt

Meyerhoff, M.

Meza‐Herrera, C. A.

Mgode, Georgies F.

Michel, Anita

Miglio, A.

MikuÅ a, Robert

Mikulski, D.

Milanesi, Marco

Milanova, Aneliya

Miller, Dave

Minato, Simone

Mioni, Mateus

Miranda, Jorge

Mirnejad, Reza

Mirshokraee, Pezhman

Mogas, Teresa

Mohamanden, W

Mohammadabadi, Mohammadreza

Mohammadi, Gholamreza

Mohebalian, Hadi

Mohebi, Reza

Mohri, Mehrdad

Molla, Wassie

Momtaz, Hassan

Monaco, Federica

Moneim, Ahmed E. Abdel

Monistero, Valentina

Monti, Gustavo

Moons, Christel

Moorman, Valerie

Moosavian, Hamidreza

Mor, S.

Moraes, Elenice A.

Morales‐Erasto, V.

Morandi, Benedetto

Moreau, M.

Moreno‐García, Miguel

Morgaz, Juan

Morrell, Jane

Mosallanejad, Bahman

Mosleh, N

Mouotailler, Sara

Mohammed MTA

Mudge, Margaret

Müller, Thomas

Mullin, Christine

Münnich, Andrea

Murakami, Tomoaki

Murtaza, Ghulam

Musallam, Imadidden

Muscher‐Banse, A

Musinguzi Simon Peter

Musser, Margaret L.

Mutascio, Liliana

Mutuku, Martin

Najera, Fernando

Namazi, Fatemeh

Naseer, Zahid

Nash, Katherine

Nasiri, Davood

Nasrollahi, Sayyed Mahmoud

Navarro, Daniela Maria do Amaral Ferraz

Naziri, Zahra

Nedi, Teshome

Nemser, Sarah

Newbrook, Kerry

Nguyen, Viet Linh

Niasari‐Naslaji, Amir

Nikbakht, Gholamreza

Niu, Bing

Njeumi, Felix

Noda, Masashi

Nofouzi, katayoun

Nolte, Wietje

Nosrati, Maryam

Novosad, Andrew

Nowakiewicz, Aneta

Ntirandekura, Jean Bosco

Obeidat, B.S.

Ocholi, Reuben A.

Ohmi, A.

Oke, Oyegunle Emmanuel

Okwumabua, Ogi

Olatunji, Opeyemi Joshua

Oliveira, M.

Ollivett, T. L.

Onal, Ahmet Refik

Osek, Jacek

Osorio, Fernando

Osoro, Eric

Oster, Henrik

Oubiña, Gonzalo

Overton, T. R.

Ozubek, Sezayi

Paduch, Jan‐Hendrik

Palmieri, V

Palomar, Ana M.

Pan, Xiaojin

Papich, Mark G.

Partovi, Razieh

Pas, Marinus F. W. te

Pathirana, Indunil

Patterson, E.

Paul, A.

Paul, Joydeep

Pedraza, F.

Pelkonen, Olavi

Pellett, Sabine

Peng, Yun‐ru

Peralta, Oscar A.

Pérez Alenza, María Dolores

Perez‐Gil, Jesus

Perkins, A.

Persson, Ylva

Pettersson, Emelie

Pfau, Thilo

Pickles, Kirstie

Pierson, Audrey

Poirel, Laurent

Poirier, Valerie

Ponnampalam, Eric

Poorghasemi, Mohammadreza

Pöppl, Alan Gomes

Posthaus, Horst

Pourjafar, Mehrdad

Pradhan, C.

Pratschke, K.

Pratt, Stefanie

Prescott, J. F.

Priviero, Fernanda B. M.

Psareva, EK

Pumphrey, S.

Punyapornwithaya, Verasak

Pye, C.

Pypendop, Bruno H.

Qian, Kun

Queiroga, Felisbina

Quinton, Helene

Radlinsky, Maryann

Raeghi, Saber

Ragab, Eman

Rahal, Sheila

Rahman, Dr. A. K. M. Anisur

Rahman, Md. Masudur

Rahman, Md. Tanvir

Ramalho‐Santos, Joao

Ramesha, Kerekoppa

Ramirez‐Valverde, Rodolfo

Ramirez, Hugo

Ramkumar, Kunka Mohanram

Raoofi, Amir

Raza, Sayed Haidar Abbas

Razijalali, Mohammad

Reagan, K.

Regnault, Sophie

Rego, Anibal

Reinhart, Jennifer

Rendle, David

Monica Isabel De Los Reyes

Rezaei, Mansour

Rezaeipour, Vahid

Riggs, Christopher M.

Rijkenhuizen, Astrid

Risselada, Marije

Rivelli, Ines

Roberts, Marilyn C.

Robertson, S. M.

Dereje Tulu Robi

Rodolakis, Annie

Rodriguez Alonso, Beatriz

Rogers, Phil

Rohdin, Cecilia

Romero, MP

Roodbari, Zahra

Roura, Xavier

Rouzbehan, Yousef

Roy, Vikas Kumar

Rucinsky, Renee

Rupprecht, Charles E.

Ryan, Michael P

Sabiza, Soroush

Sadaka, Dr. Ayman

Safarpoor, Farhad

Sagane, Yoshimasa

Saif, Majid

Sakhaee, Ehsanollah

Sakurai, Yoshihiko

Salci, Hakan

Saleh, Ahmed

Saleh, Meriam

Salem, Shebl

Salmani, Fatemeh

Sambo, James

Samir, Haney

Sanchez‐Calabuig, MJ

Sanchez, Samuel

Santoro, Domenico

Saravana Kumar, Pachaiyappan

Sarkar, S

Sarotti, Diego

Sattar, Sadia

Sauter‐Louis, Carola

Sazmand, Alireza

Schembr, Mark

Schkeeper, Amy

Lindley, Stephanie

Schmidt, Volker

Schmiedt, Chad Weber

Schmoelzl, Sabine

Schnee, Christiane

Schreiter, Ruben

Schulz, Katja

Schusser, Gerald‐Fritz

Schuyler, Qamar

Sdiri, Chaïma

Sechman, Andrzej

Seidavi, Alireaza

Seller, S.

Selmi, Houcine

Selmic, Laura

Seo, Kyoung won

Seo, Min

Sergiel, Agnieszka

Seyboldt, Christian

Seyoum, Zewdu

Shahardar, R. A.

Shamsi, Shokoofeh

Sharifi, Iraj

Sharma, Devina

Sharma, Hari P.

Sharma, Rekha

Sharp, C.

Shawaf, Turke

Shewmaker, David

Shokoohi, Majid

Shuaib, Muhammad

Shyaka, Anselme

Siddiki, Amam Zonaed

Silverstein, Deborah

Singh, Brij Pal

Singh, Preet

Skinner, O.

Slapeta, Jan

Sluiter, Kristi

Smith, M. Ryan

Smith, Sionagh

Snoeck, Chantal

Snyder, Lindsey Beth Culp

Soleimanzadeh, Ali

Song, Woo‐Jin

Sonne, Luciana

Sorden, Steven

Sorror, Wafaa

Soto‐Barrientos, N

Soto, Sara

Souza, Wildelfrancys

Sriniyasan, Subramani

Srisook, Klaokwan

Srivastava, N

Stankovic, Milan

Stefanon, Bruno

Sting, Reinhard

Stingl, K

Stram, Yehuda

Stucki, Dimitri

Studer, Eveline

Su, You‐Qiang

Sukparangsi, Woranop

Sun, Hao Yang

Sung, Kidon

Suter, S.

Suzuki, Tohru

Swelum, Ayman Abdel‐Aziz

Szymanska‐Czerwinska, Monika

Tadesse, Belege

Tallon, Rose

Talluri, T. R.

Tamrat, Habtamu

Tanaka, Noriko

Tantia, M. S.

Tarekegn, Zewdu Seyoum

Tartor, Y

Tauro, Anna

Tavassoli, Mousa

Tehrani Sharif, Maysam

Tellez, Guillermo

Tenhagen, Bernd‐Alois

Tetens, Jens

Thanh, Lam Phuoc

Tho Seeth, Martin

Thomas, Prasad

Thompson, Peter N.

Thomson, Katariina

Thongsahuan, S.

Thongwat, Damrongpan

Tian, Rugang

Toghyani, Majid

Tolone, Marco

Tolosa, Tadele

Tomanovic, Snezana

Tomaso, Herbert

Tomczuk, Krzysztof

Tootian, Zahra

Torki, Mehran

Toutain, P. ‐L.

Trees, Eija

Tremolada, Giovanni

Tsuka, Takeshi

Uddin, Md Bashir

Udraite, L.

Uehlinger, F.D

Uemura, Akiko

Ulbert, Sebastian

Uney, Kamil

Van Asselt, N.

Van Der Drift, Saskia

Van Heerden, Henriette

Vannucchi, C. I.

Vasco, Karla

Véliz, Francisco

Verstraete, F.J.M.

Vieira Neto, A

Villot, C

Vizoná, Rafael

Vogt, Isabelle

Vogt, Nadine

vojdani, zahra

Vollmar, Andrea

von Schweinitz, Dietrich

Vounba, Passoret

Vrbanac, Zoran

Wagner, Henrik

Walker, Lin

Walmsley, Darby

Wanapat, Metha

Wang, Jicang

Wang, Leyi

Wang, Liansheng

Wani, Z. A.

Wardrop, K. J.

Wasyl, Dariusz

Wavreille, V.

Weber, Jim

Wei, Ren

Weishaar, Kristen

Wernery, U

Wesselowski, Sonya

White, Steven

Whitfield, Chase T.

Wibowo, Michael

Wilkens, Mirja

Willemsen, A.

Williams, David

Wills, Alison

Winslow, Christine

Withers, Sita

Witter, Kirsti

Woclawek‐Potocka, Izabela

Wood, R. Darren

Wu, Bangyuan

Wu, Wenxuan

Xiao, Liu

Xin, Yin

Xu, Hong

Xu, Rifu

Xu, Shiwen

Yadav, Sarvajeet

Yang, Fan

Yangchun, Cao

Yildiz, K

Yoder, Peter

Youn, Hwa‐Young

Youssef, Ibrahim M. I.

Yu, DoHyeon

Yuan, Xiaoyuan

Yue, Min

Zaineldin, Amr I.

Zajac, Magdalena

Zhang, Weiyu

zhang, ji

Zhang, Jianhai

Zhang, K

Zhang, Keyu

Zhang, Tao

Zhang, X

Zhou, Z.

Zhu, FenXia

Zohorul Islam, Md

Zokaei, Maryam

Zou, Likou

Zur Linden, Alex

